# Combinations of radiotherapy with immunotherapy in cervical cancer

**DOI:** 10.7150/jca.65074

**Published:** 2022-02-28

**Authors:** Xiaojing Yang, Hanru Ren, Jie Fu

**Affiliations:** 1 Department of Radiation Oncology, Shanghai Jiao Tong University Affiliated Sixth People's Hospital, No. 600, Yishan Road, Shanghai, 200233, China.; 2 Department of Orthopedics, Shanghai Pudong Hospital, Fudan University, Pudong Medical Center, Shanghai 201300, P.R China.

**Keywords:** Immunotherapy, Radiotherapy, cervical cancer

## Abstract

Immunotherapy serves as another effective cancer treatment apart from surgery, chemoradiotherapy, and targeted drug therapy. Radiotherapy combined with immunotherapy has significantly improved the effective cure rate for patients in several clinical trials. It subverted the traditional view that radiotherapy kills immune cells and has immunosuppressive effects, indicating a synergistic effect of radiotherapy and immunotherapy. In this article, we reviewed and summarized the molecular mechanism of the combined use of radiotherapy and immunotherapy, as well as the clinical treatment and safety of the combination of the two. We describe the rationale for the integration of radiotherapy and immunotherapy in patients with cervical cancer, present safety and efficacy data that support this combination strategy, and highlight unanswered question sand future research needs. Besides, this study can be referenced for clinicians to guide subsequent clinical medicine.

## Introduction

In 2018, there were 569,847 new cases of cervical cancer and 311,365 deaths worldwide, ranking the fourth in female malignant tumors in terms of both incidence and mortality 1. Due to the popularization of cervical cancer screening and the promotion of HPV vaccine, cervical cancer has largely become a preventable disease, however, its 5-year survival rate is still only about 60% 2. For early cervical cancer without metastasis, surgery and chemoradiotherapy are the main treatments, and the 5-year survival rate can reach 88% to 95% in patients with FIGO stage IB to IIA without lymph node metastasis. However, for metastatic or recurrent cervical cancer, traditional treatment methods have not achieved satisfactory results 3. With the use of targeted therapy and immunotherapeutic drugs, the survival time of these patients has been significantly prolonged, but the final curative effect has not been achieved, and more in-depth molecular level studies are urgently needed to reveal new therapeutic targets to guide clinical individualized treatment.

Immunotherapy serves as an effective cancer treatment in addition to surgery, chemoradiotherapy, and targeted drug therapy. Immune checkpoint inhibitors (ICIs) are one of the most successful treatments drugs. They have been used to treat numerous cancers including melanoma, Hodgkin's lymphoma, and bladder cancer due to their definitive efficacy and long-term response. ICIs such as CTLA-4-mediated irinotecan or PD-1 (programmed cell death-1)/PD-L1 (programmed cell death-ligand -1) axis inhibitors act by blocking specific interactions between dendritic cells and T cells 4. PD-1/PD-L1 inhibitors specifically block the interaction between effector T cells and cancer cells and/or dendritic cells, which usually leads to the inhibition of T cell growth as well as the loss of effector function 5. PD-1 and PD-L l inhibitors have been approved by the US Food and Drug Administration for clinical use, and the China Food and Drug Administration has clinically approved many ICIs. The combination of immunotherapy and radiotherapy has become a popular research field after the publication of the PACIFIC 6-8 and PEMBRO-RT studies 9-10.

Compared to immunotherapy, radioimmunotherapy has significantly improved the effective cure rate for patients in a number of clinical trials. It subverted the traditional view that radiotherapy kills immune cells and has immunosuppressive effects, indicating a synergistic effect of radiotherapy and immunotherapy 11. The literature has reported a patient with advanced cervical cancer who received only pelvic radiotherapy for economic reasons, but her para-aortic lymph nodes shrank significantly after treatment 12. Radiotherapy can affect the tumors' immun state in a variety of ways, such as promoting the release of tumor-specific antigens by tumor cells and improving the killing ability of the immune system 13. Meanwhile, the introduction of immunotherapy may further promote this series of processes, and even increase the incidence of “abscopal effect” 14.

## The immunotherapy for cervical cancer

The mechanism of immunotherapy for cervical cancer is mainly to activate the human immune system and rely on autoimmunity to kill cancer cells and tumor tissues. The interrelationship between tumors and the immune system can be divided into three different stages. In the clearance phase, the nascent tumor is immunogenic and can be recognized by the host's innate and adaptive immune systems and removed. A small proportion of surviving tumor cells have weakened their own antigenicity and evade the clearance function of the immune system and enter the second stage - equilibrium stage, when tumor cells are still under the clearance pressure of the immune system and cannot overgrow. This balance is broken when mutations in tumor genes are involved to a certain extent, that is, they enter the escape stage and produce a series of malignant phenotypes 15. Therefore, the key to cancer immunotherapy is to remodel the lasting and effective anti-tumor immune response, including immune checkpoint inhibitors, therapeutic vaccines, tumor-infiltrating T cell therapy, etc. These three therapies have different mechanisms of action and advantages and disadvantages in the treatment of cervical cancer (Table 1).

## Immune checkpoint inhibitors

Immune checkpoints refer to regulatory molecules present in the immune system and are mainly expressed on the surface of immune cells. According to function, it can be divided into costimulatory immune checkpoint and inhibitory immune checkpoint. Current research has focused on inhibitory immune checkpoints, including cytotoxic T lymphocyte antigen 4, programmed death protein 1, and their ligands.

### Anti-CTLA-4 monoclonal antibody

Lheureux S et al. 16 reported that the results of the clinical trial study of anti-CTLA-4 monoclonal antibody ipilimumab for metastatic or recurrent cervical cancer showed that 42 patients with cervical cancer previously treated with radiotherapy or chemotherapy received ipilimumab, of the 34 evaluable patients, 1 had partial response, 10 had stable disease, 23 had progressive disease. The results of the study showed that the anti-CTLA-4 monoclonal antibody ipilimumab had some efficacy for metastatic or recurrent cervical cancer.

### Anti-PD-1 monoclonal antibody

Pembrolizumab is a humanized monoclonal immunoglobulin G4 antibody that targets PD-1. Frenel JS et al. 17 reported that the results of the multicenter clinical trial of the safety and efficacy of anti-PD-1 monoclonal antibody-pembrolizumab in patients with PD-1-positive advanced solid tumors showed that among 24 patients with PD-1-positive advanced cervical cancer who had failed previous treatment, 4 had partial response, with an overall response of 17% and 3 had stable disease. In June 2018, pembrolizumab was approved for the treatment of patients with recurrent or metastatic cervical cancer that has progressed and whose tumors express PD-L1. Recently, a supplementary report from the N1 study showed that 98 patients with recurrent or metastatic cervical cancer were treated with pembrolizumab with a follow-up time of 10.2 months. The ORR was 12.2%, including 3 cases of CR and 9 cases of PR 17. Based on the results of this study, pembrolizumab is recommended in the 2020 NCCN guidelines for cervical cancer as a second-line regimen for recurrent cervical cancer indicated for PD-L1-positive or MSI-H/dMMR.

Nivolumab is a humanized monoclonal antibody directed against the PD-1 receptor. The NCT02488759 trial 18 included 19 patients with recurrent or metastatic cervical cancer who were given intravenous navulizumab every 2 weeks, and the data showed that the ORR of 19 patients with cervical cancer was 26.4%, with no treatment-related deaths. It can be seen that nivolumab showed good safety and antitumor activity in the treatment of patients with advanced or recurrent cervical cancer. However, a recent phase II clinical trial reported that the investigators included 26 patients with relapsed and refractory cervical cancer after platinum-based chemotherapy who were treated with intravenous nafulizumab every 2 weeks 19. The results showed that among 26 patients with relapsed and refractory cervical cancer who were followed up for 32 months, 21 patients experienced treatment-related adverse events (TRAEs), most of which were grade 1-2. Six patients developed grade 3 TRAEs, and 1 patient discontinued the use of nivolumab due to hepatotoxicity. No Grade 5 TRAEs occurred, and 2 patients had Grade 5 TRAEs. One patient had PR with a response time of 3.8 months. SD occurred in 36% of patients, and the duration of SD was 5.7 months. From the data, it can be seen that single-agent nivolumab has low antitumor activity and acceptable safety in patients with relapsed and refractory cervical cancer.

### Anti-CTLA-4 monoclonal antibody combined with anti-PD-1 monoclonal antibody

In addition to the immune checkpoint study, recently, Naumann RW et al.^20^ reported the interim results of the 358 trial, in which patients with recurrent or metastatic cervical cancer were randomly divided into two groups: nivolumab 3 mg/kg q2w + ipilimumab 1 mg/kg q6w (group A) or nivolumab 1 mg/kg + ipilimumab 3 mg/kg q3w for 4 doses, followed by nivolumab 240 mg q2w (group B) for 24 months until progression or unacceptable toxicity, the primary assessment indicator was ORR, and the secondary assessment indicators were OS, PFS and duration of response. The results suggested that: both regimens had clinical efficacy in recurrent and metastatic cervical cancer; PD-L1 response could be observed regardless of tumor cells; patients who had not received systemic treatment were observed to have better efficacy with the two regimens; follow-up time was > 10 months, and the response was durable; no new adverse reactions occurred, and the safety of the treatment regimen was controllable.

### Adoptive T cell therapy

Adoptive T cell therapy refers to the therapy in which autologous or allogeneic tumor-specific T cells are expanded *in vitro* and reinfused into patients to kill tumors, which mainly includes tumor-infiltrating T cells, T cell receptor-modified T cells, and chimeric antigen receptor T cells. Compared with peripheral cells, tumor-infiltrating T cells have a higher proportion of tumor-specific T cells, which can be greatly expanded and show tumoricidal effect after interleukin-2 stimulation *in vitro* 2, 21. The number of tumor-specific T cells obtained by this method is much more than that obtained by therapeutic vaccines, so it has received much attention in adoptive cell therapy. NCT03108495 is a phase 2 clinical trial assessing the safety and efficacy of TIL in patients with recurrent metastatic cervical cancer. After 9 patients were treated with HPV-TILs, 2 patients achieved CR and 1 patient achieved PR 22. Treatment-related adverse reactions are mainly hematology-related toxicities caused by lymphocyte-clearing chemotherapy, such as anemia and lymphopenia. This trial shows the reliable efficacy and safety of adoptive T cell therapy in advanced cervical cancer and is worthy of more in-depth study.

### Therapeutic vaccines

Therapeutic vaccines activate cytotoxic T cells to specifically kill tumor cells by introducing various forms of tumor antigens, such as tumor cells, tumor-associated peptides, or genes expressing tumor-specific antibodies, into patients 23. Therapeutic vaccines are characterized by high immunogenicity and can trigger strong and persistent humoral and cellular immunity, but there are also some urgent problems to be solved, such as the potential risk of treatment, especially for immunocompromised patients, and the immune response produced by the body will become weaker after repeated treatment with the same carrier 24. In cervical cancer, the host cells infected with HPV express viral proteins E6 and E7 sustainably and may be ideal antigens for therapeutic vaccines in cervical cancer. Among the many therapeutic vaccines, live vector vaccines have attracted much attention due to their high immunogenicity. Listeria monocytogenes (Lm) is a negative bacterium that escapes the lysis of lysosomes and proliferates within antigen-presenting cells to trigger strong innate and adaptive immune responses 25. ADXS11-001 is a live attenuated vaccine for Lm that secretes HPV-16E7 antigen bound to a non-hemolytic fragment of Lm hemolysin O (LLO). In the phase I clinical trial NCT02853604, 15 patients with recurrent metastatic cervical cancer were included in the study. After receiving the vaccine, all patients developed symptoms of influenza and six developed grade 3 adverse events, but no grade 4 adverse events occurred. After the end of the trial, two patients died, five had progressive disease, seven had stable disease, and one patient achieved a partial response, and this trial demonstrated for the first time the safety of attenuated active Lm in patients with advanced cervical cancer 26.

## Preclinical study of immunotherapy combined with radiotherapy

Numerous preclinical studies have reported that the use of CTLA-4 inhibitors improves radiotherapy efficacy and local response 27-29. A retrospective case study consisting of 101 patients who were treated with ipilimumab confirmed this improvement. 70 out of 101 patients received both radiotherapy and immunotherapy. Compared with those patients who only accept immunotherapy, patients who accept both treatments had a significant increase in overall survival rate as well as an improved response to treatment 30. The interaction of PD-1/PD-L1 inhibition and radiation therapy has also been reported to enhance the local and distant efficacy 31-33. In addition, another reason for the combination of the PD-1, CTLA-4, and radiotherapy is that both PD-1 and CTLA-4 activate non-redundant immune mechanisms 34. IL2 is a cytokine that plays an important role in the activation of immune responses. Although it stimulates the proliferation of regulatory T cells, it also activates T cell cytotoxicity and natural killer (NK) cells. This mechanism activates the immune system and increases the local response to immunoradiotherapy 35. These “immune cytokines” include NHS-IL2 and L19-IL2, etc. 36-38. L19-IL2 only works in EDB-expressing tumors, which largely depends on the presence of CD81 cytotoxic T cells 39, 40. However, even in tumor models lacking the MHC I class, the cytotoxicity of cancer cells does not depend on the specific antigen-targeting activity of CD81T cells; instead, it depends on the activity of NK cells, where radiotherapy alone has an additive effect on L19-IL2 41. Moreover, it is reported that the combination of radiotherapy and L19-IL2 also has a significant therapeutic effect on secondary tumors.

Clinical trials of radiotherapy in combination with CTLA-4 inhibitors, PD-1/PD-L1 inhibitors, vaccination or cytokines (e.g., IL2), anti-transforming growth factor-β, and granulocyte-macrophage colony-stimulating factor are in progress 42-44.

## Mechanism of action of radiotherapy combined with immunotherapy

**Figure 1:**
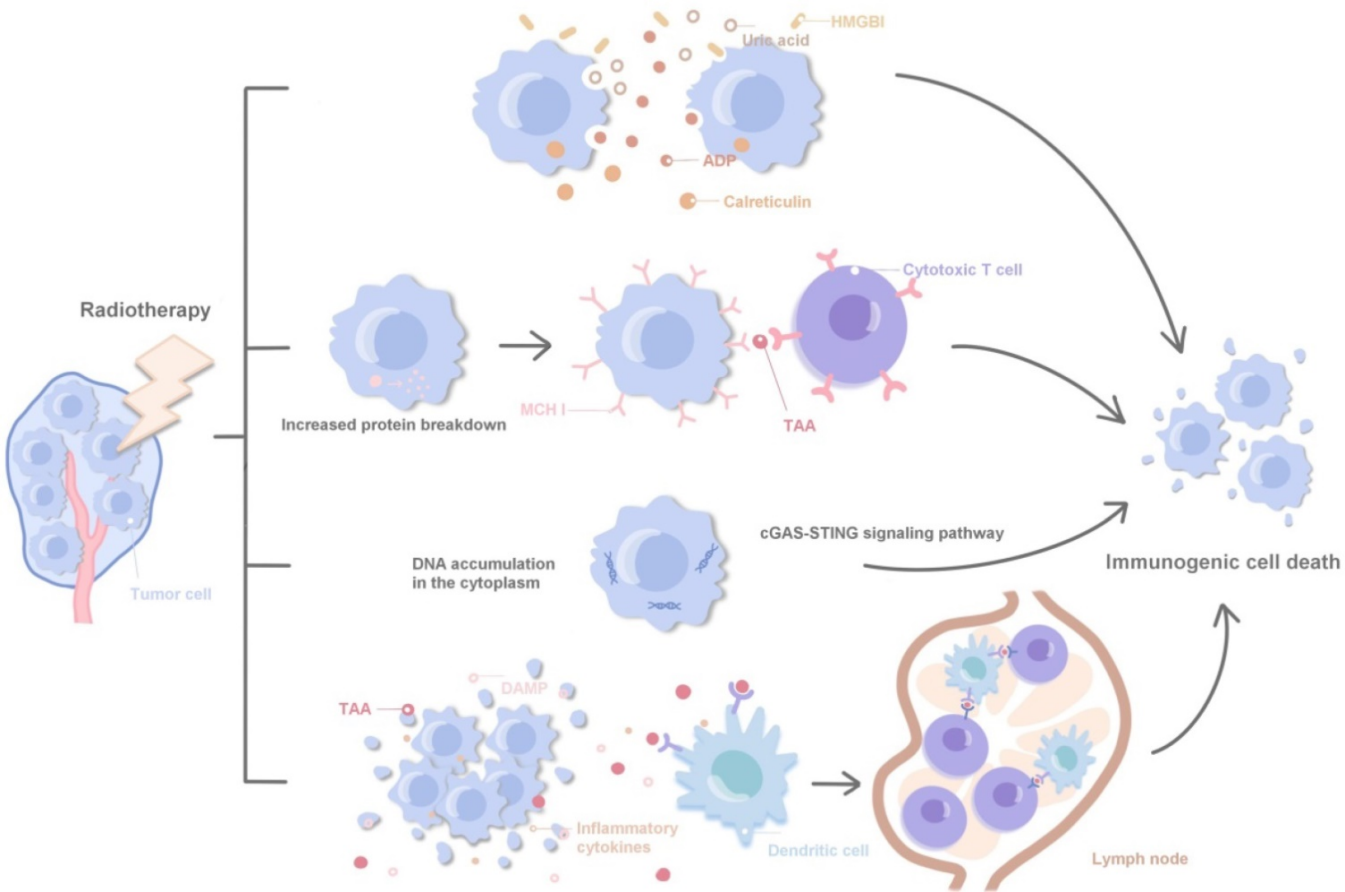
Related molecular mechanisms of radiotherapy combined with immunotherapy.

Radiotherapy can achieve a synergistic effect with immunotherapy by directly inducing the immunogenic death of tumor cells, regulating tumor cell phenotype, normalizing tumor vessels and promoting immune cell infiltration and local infiltration of systemic therapeutic drugs 11, 45, 46. Specifically, ① Radiotherapy can promote the release of HMGBl, ADP and uric acid, and promote the immunogenic death of tumor cells by stimulating calreticulin transport to the cell surface; ② Radiotherapy leads to increased protein decomposition, induces the increased loading and expression of MHCI protein on the surface of tumor cells, and promotes cytotoxic T cells in recognizing tumor-associated antigens; ③ Radiotherapy promotes cytoplasmic DNA accumulation and induces immune activation via agonists of the STING and cGAS pathways; ④ DAMPs, TAAs and inflammatory cytokines in cell debris released after tumor death activate antigen-presenting cells, such as dendritic cells, and present TAAs to immune cells in lymph nodes. Polyclonal TAAs-specific cytotoxic T cells are activated and kill irradiated local and distant tumors. Accordingly, immunotherapy enhances this response, laying a foundation for the combination of radiotherapy and immunotherapy (Fig. 1). Local radiotherapy can activate the immune system and trigger immune cells to attack tumor cells far away from the irradiated area, which is called 'abscopal effect'. In clinic, radiotherapy itself rarely produces abscopal effect, but immunotherapy can enhance the immune induction effect of radiotherapy and increase the incidence of abscopal effect 47, 48. Radiotherapy and immunotherapy synergistically inhibit the tumor growth, achieving an effect of 1 + 1 > 2 49. The synergistic effect of radiotherapy and immunity can be further divided into: ① Spatial coordination: radiotherapy produces local cytotoxicity, and immunotherapy has an effect on local tumors and distant metastases; ② Time synergistic effect: radiotherapy has a rapid onset of action, effectively limits tumor progression, and provides enough time window to cope with the delayed effect of immunotherapy; immunotherapy is different from other cancer treatment methods such as chemoradiotherapy, the patient's response to immunotherapy is often delayed, and even the tumor burden will be temporarily increased during immunotherapy, and the rapid radiotherapy effect plays a good complementary role; ③ Biological synergistic effect: radiotherapy, chemotherapy and immunotherapy are aimed at different cell populations, respectively, which produces a synergistic effect in biology.

## Integrating radiotherapy and immunotherapy into the clinic

Several factors should be considered during the integration and optimization of immunotherapy and radiation therapy: Optimal fractionation dose and total dose, overall scheduling between of radiotherapy and immunotherapy, choice of radiotherapy technique, extent of CTV, and safety issues.

The initial objective of radioimmunotherapy should be considered as follow: Does this treatment aim to improve local effects or trigger an abscopal effect on non-irradiated micro metastases? As described above, radiotherapy stimulates the immune system by expanding the immune range of T cells (vaccination effect), attracting T cells to the irradiated site and rendering irradiated cells more susceptible to T cell-mediated cell killing (vulnerability effect). As can be expected, only an expanded immune range would produce a broad range of systemic responses of the immune system, implying that the underlying biological mechanism is different. Several groups have applied different fractionation and total doses settings in their protocols respectively. Gandhiet et al. 50 described the dose-dependent increases in cell surface molecules in a dose range from 1 to 50 Gy in human HCT116 colorectal cancer, Mel JuSo melanoma cells as well as murine MC38 colon cancer cells. Since these receptors are important for the vulnerability of T cells, they were considered as the most crucial factors in enhancing the local effects of radiotherapy. Afterwards, an evaluation was carried out between fractionated (5 * 3 Gy) and single-dose (15 Gy) radiotherapy, aiming to compare the effectiveness of activating dendritic cells in lymph nodes in the B16 melanoma model, in which a single dose of 15 Gy was found to be more effective. In contrast, in a similar T cell priming B16-OVA melanoma tumor model, another group demonstrated 2 * 7.5 Gy fractionation was more effective than a 15 Gy dose fractionation 51. Several groups have also studied the effect of fractionation in radiotherapy when combined with immunotherapy. Dewan et al. 52 put forward that although all fractionation regimens exhibited comparable local tumor control compared to combined CLTA-4 inhibition, 3 * 8 Gy was found to be superior to 5 * 6 or 1 * 12 Gy in TSA breast and MCA38 colon cancer models in terms of local tumor control and absolute response. Moreover, a retrospective analysis of clinical data from melanoma patients treated with ipilimumab showed that low-dose radiotherapy less than 3 Gy was associated with absolute response 53. The interaction of L19-IL2 immunocytokine and radiotherapy in the C51 tumor model suggests that irradiation with larger doses is more effective in inducing an absolute response since only 1 * 15 Gy, rather than 5 * 5 or 5 * 2 Gy, can induce curative treatment of tumors. In summary, fractionation and dose configurations are essential in maximizing the effect of the relevant immunotherapy; however, no consensus exists as to which treatment regimen is optimal. Conflicting results in literature suggests that optimal segmentation is highly context-dependent; therefore, conclusions should be drawn with caution. As tumor response depends on the schedule of radiation therapy as well as factors within the cancer immune cycle, the impact of choosing the correct fractionation schedule when investigating local or absolute tumor control will be diminished. Therefore, assessing the efficiency of different fractionation protocols with more direct methods may have to rely on innovative biomarkers, such as the release of chemokines and cytokines associated with immunogenic cell death in blood or biopsies 54, or screening > 1000 T cell specificities in a single sample. This approach may enable the comparison of the impact and efficiency of different radiotherapy regimens on increasing the diversity of specific T cell responses against different tumor antigens.

Combined with our own experience in clinical treatment, we believe that high-dose fractionated radiotherapy can induce immunogenic cell death and conventional fractionation can induce M1TAM, allowing T cells to infiltrate the tumor. Therefore, multiple fractionations are superior to single fractionation; 7-8 Gy may induce immunogenic cell death. Up to more than 12-18 Gy, it degrades DNA and does not produce DNA fragments, which reduces immunogenicity. Immune promotion and immunosuppression coexist, and how to select the appropriate dose and fractionation is difficult. The dose fractionation is applicable to different tumors.

Radiotherapy techniques such as Volumetric Modulated Arc Therapy can better fit the CTV; however, it also supplies low-dose radiation to most body tissues 55. Lymphocytes are the most radiosensitive cells in the human body, and their D10 is only 3 Gy. In this case, the dose delivered to the lymph nodes as well as the fractionations are important. The normal transition time of naive cytotoxic T lymphocytes ranges from 12 hours to one day 56. Nevertheless, when faced with antigen presentation by dendritic cells, these T cells remain in contact with dendritic cells and undergo "explosive" transformation. This process again takes 24 h, even before the occurrence of clonal expansion 57. However, for cytotoxic T cells, stable interact with dendritic cells is essential and still permit successful effector differentiation, though their long-life memory is hampered 58. Even small doses of lymph nodes with very short daily intervals may interfere with the priming process of T lymphocytes and their memory function. Till now, the effects of low-dose irradiation and daily fractionation are unknown and thus require further investigation.

The integration of radiotherapy in immunotherapy protocols may also require physicians to think twice about the scope of CTV. For example, when radiotherapy is introduced to an immunotherapeutic patient suffering from metastatic disease, expansion of the CTV may not be necessary as it may be sufficient to irradiate a portion of the tumor in order to induce immune stimulation. A narrower margin would better protect the OAR, thus reducing complications. Theoretically, this approach is promising but requires further validation in clinical trials.

Till now, the optimized selection method of the correct CTV range in combination with immunotherapy for triggering abscopal effects remains unclear 59. Accordingly, various authors have proposed many mathematical models for its prediction, which is based on the trafficking of T cells and the assumption that absolute effects can only be achieved when a sufficient number of activated T cells from irradiated tumors were able to reach distant sites. However, since no clinical data are available to validate this virtual model, extra care should be taken prior to utilizing the model, as it lacks many other important parameters to determine the systemic response 60.

Radiotherapy produces immune antigens and activates T cells, but it also produces immunosuppressive expression, such as the expression of PD1 and PD-L1. At this time, the addition of anti-PD-L1 therapy can increase the aggression of T cells, improve the quality and quantity of T cells, and is more conducive to the treatment of primary and metastatic tumors. Timing seems to be crucial in the design of immunoradiotherapy protocal in order to attain optimal results 61. The ideal timing between immunotherapy and radiotherapy depends on the mechanism of action of specific forms of immunotherapy. For example, Young et al. investigated the optimal timing of radiotherapy in combination with OX-40 agonist antibody and CLTA-4 antagonist antibody in which administration of CTLA-4 prior to giving radiotherapy was found to be the best. Nevertheless, other authors have found synergy with concurrent or sequential administration of CTLA-4 inhibitors with radiotherapy 52, 62. In contrast, OX-40-based immunotherapy was noted to best be performed immediately after radiotherapy 61. The authors proposed that OX-40 functions by increasing antigen-specific T cell numbers, while anti-CTLA-4 acts as a down-regulator of regulatory T cells 63. Thus, inhibition of OX40 is most beneficial only after radiation-induced antigen release. In regard to CTLA-4 inhibition, antigen release produced by radiation therapy is most effective only when the regulatory T cell grade is initially removed. Dovedi et al. 64 studied the ideal sequence of treatment so as to inhibit the PD-1 axis. The best results were obtained when PD-L1 was administered simultaneously or immediately following radiotherapy. Delay of PD-L1 infusion by 1 week abolished the interaction between radiotherapy and PD-1 axis inhibition 65. Moreover, inhibition of the PD-1 axis increases the lytic activity of cytotoxic T cells 5. Thus, the best interaction can be expected only at the moment where radiotherapy temporarily induces surface ligands on cancer cells to increase their vulnerability to T cell attack 66. The authors also demonstrated that radiotherapy temporarily induced the overexpression of PD-1 axis molecules on tumor cells and T cells infiltrating the tumor 64. Thus, the inhibition of the PD-1 axis is closely related in the time of radiation treatment. Therefore, inhibition is expected to be most effective when it best attenuates radiation-induced immune responses.

Hypoxia is associated with radioresistance and immunosuppression, which can be quantified using anoxic positron emission tomography tracers 67. Understanding these mechanisms of resistance related to hypoxia may identify new therapeutic targets 68. In addition, the use of hypoxia-targeting agents to reduce hypoxia may result in a decrease in immunosuppression in the tumor microenvironment 69.

Finally, there is only a limited amount of clinical data of different combinations of immunotherapy and radiotherapy. Immunotherapy, upon activation of the immune system, may exacerbate acute radiation-induced toxicity associated with the inflammatory response 70. Kroezeet al. 71 recently reviewed stereotactic radiotherapy as well as studies that utilized both anti-CTLA4 and anti-PD-1/PD-L1. Here, the combination of anti-CTLA4 and cranial stereotactic radiotherapy was observed to be safe. Very limited data are available in regard to the combination of extracranial stereotactic radiotherapy with anti-CTLA4. In light of the combination of simultaneous anti-PD-1/PD-L1, the authors claimed that clinical data is not sufficient enough for drawing the corresponding conclusions. Following this report, Levy et al. 72 published a small phase I/II trial that investigated the PD-L1 inhibitor durvalumab in combination with conventional stereotactic radiotherapy, which was found to be well tolerated. Kwon et al. evaluated the combination of 8-Gy conventional radiotherapy with eplerenumab versus placebo in a large phase III clinical trial that consisted of patients with metastatic castration-resistant prostate cancer. Although the primary endpoint (overall survival) was negative, they did not find that the radiotherapy-ipilimumab combination was more toxic than ipilimumab. Additionally, two small phase I clinical trials have shown that NHS-IL2- or IL2-based immunotherapy can be safely performed following conventional or high-dose stereotactic radiation therapy, respectively 36, 73. In summary, the available data suggest that radioimmunotherapy does not confer greater toxicity than immunotherapy alone. However, since the corresponding data is limited, it is recommended that these combinations be preferably tested within the context of a clinical trial.

## Safety of radiotherapy combined with immunotherapy

Based on the clinical evidence above, the future of immunoradiotherapy is highly promising. However, whether this combined therapy model increases the incidence of adverse reactions above grade 3 would determine its feasibility in clinical practice. As ICIs become a part of cancer treatment, clinicians have gradually gained more experience and a better understanding of treatment-related adverse reactions/immune-related toxicity. However, till now, the clear pathological mechanism for the occurrence of immune-related adverse effects (irAEs) is still a challenge for researchers. Possible mechanisms including immunotherapy could improve the response of T cells to autoantigens expressed in normal tissues, increasing the expression of autoimmune antibodies, secretion and release of immune factors, and complement-mediated immune responses 74. As an immune checkpoint of the target, cytotoxic T lymphocyte associated antigen-4 (CTLA-4)/PD-1 is expressed in normal tissues and participates in the formation of immune homeostasis, such as CTLA-4, which is expressed on the pituitary gland. The mechanisms by which radiotherapy and ICls synergistically modulate immune responses may also influence the type and severity of treatment-related adverse effects. Data from a series of retrospective studies as well as a few prospective single-arm/randomized studies have provided substantial evidence that the combination of palliative radiotherapy and ICls is generally safe and does not confer substantial increases in immune-specific adverse events 75. In addition, existing evidence suggests that the use of PD-L1/PD-1 inhibitors does not increase the incidence of Grade 3 pneumonitis after curative intent chemoradiation for Stage III NSCLC. However, reports suggest that the use of ICls in brain metastases patients treated with high-dose stereotactic intracranial radiation may increase the risk of treatment-related brain necrosis. Compared to delivering immunotherapy and radiotherapy solely, a number of clinical trials have suggested that radiotherapy combined with immunotherapy does not significantly increase grade 3 or higher adverse reactions. However, whether the adverse reactions of radiotherapy combined with immunotherapy will become a bottleneck in its furthur clinical application is still a significant topic of concern.

No clear evidence shows that radiotherapy combined with immunotherapy could lead to an increased incidence of adverse reactions, and the toxicity was within the tolerable range. Concurrent chemoradiotherapy followed by sequential immunotherapy is a new standard treatment modality for inoperable stage III NSCLC, and concurrent chemoradiotherapy combined with immunotherapy can significantly prolong the patients' survival expectations based on evidence from current randomized phase III controlled clinical studies. As clinical evidence continues to increase, immunotherapy timing may gradually advance from patients in stages III and IV to those with early-stage tumors. One of the recent research trend is to improve the efficacy of radiotherapy combined with immunotherapy and reduce adverse reactions in the early stage of concurrent chemoradiotherapy, and to develop new combine treatment protocols for early-stage cancer patients. Another important research frontier is the introducingICIs into immunoradiotherapy, which is promising to be a candidate for replacing the chemotherapy.

## Thoughts on brachytherapy combined with immunotherapy

For the treatment of cervical cancer, there are brachytherapy methods. The possible advantages of brachytherapy on immune regulation are as follows: compared with external beam radiation, it has less damage to systemic lymphocytes; provides the superposition of different doses; multi-fractionation therapy provides attack time for immune cells; has less dose to normal tissues, even if it has little effect on the subsequent treatment after failure; has a large dose drop gradient, has less damage to infiltrating immune cells around the tumor, and the surrounding low-dose area is often the recurrence area of plugged brachytherapy. So, can only partial irradiation produce sufficient immune promotion? Can brachytherapy be used to give partial tumor radiotherapy and minimize the normal tissue dose (the total rectovesical dose limit is less than 10 Gy); brachytherapy produces a continuous dose gradient from 0.5 Gy-10 Gy above, can ICD be produced while minimizing the damage to tumor-infiltrating immune cells? 2-5 days after radiotherapy is the time for immune cells to recruit and activate into the tumor. Whether it is necessary to avoid it is a time for the next treatment, such as weekly radiotherapy? These issues need to be further investigated by us.

## Summary and future prospects

The combination of radiotherapy and immunotherapy is safe and controllable with clear efficacy. Although there are still many controversial problems, with the increasing clinical application, the controversies may gradually be resolved, and other problems may appear. The evolutions of clinical applications and technological advances have maximized the activation and initiation of immunity and have reduced treatment-related adverse reactions. The improvement of systemic treatment efficacy both prolongs the patients' survival time and increase the need for local treatment, which is complementary. Moreover, the improved efficacy of systemic therapy increases the importance of local therapy, which is further necessary to study the combination of local therapy with systemic therapy in achieving optimal results. There are still many unsettled problems hanging regarding the optimal combination between radiotherapy and immunotherapy. These problems are related to optimal fractionation, target dose, treatment technology, treatment timing and safety. A large number of clinical trials are currently underway to investigate the radio-immune interactions in patients. Although radioimmunotherapy is mainly applied to locally advanced patients at present, better clinical benefits can be obtained considering the good physical condition, better immune status and relatively small tumor burden of early patients. In the future, with the increase of clinical evidence, radioimmunotherapy will gradually have the opportunity to participate in the treatment of tumor patients at an earlier stage.

## Figures and Tables

**Table 1:** Clinical trials of immunotherapy for cervical cancer

Test name (test phase)	Medication plan	Treatment effect	Adverse effect (AE)
KEYNOTE-158 (Phase II)	200 mg pembrolizumab/3 weeks	ORR: 12.2%	Hypothyroidism (10.2%); loss of appetite (9.2%); fatigue (9.2%)
CheckMate-358 (Phase I/II)	240 mg nivolumab/2 weeks	ORR: 26.3%	AE (63.2%); AE of grade 3 and above (21.1%)
GOG 9929 (Phase I)	Ipilimumab 3mg/kg; 4 doses/21 days	1 year DFS: 74%	AE grade 3 and above: diarrhea, enteritis
CT02853604 (Phase I)	Dose 1: ADXS11-001 1×109/21 days; Dose 2: ADXS11-001 3.3×109/21 D; Dose 3: ADXS11-001 1×1010/21 D	7 cases SD; 1 case PR	Influenza symptoms (100%); Grade 3 and above AE: (40%)
NCT03108495 (Phase II)	HPV-TIL infusion; Interleukin 720 000 IU/kg/dose/8 h	7 cases CR; 1 case PR	Anemia (100%); Lymphopenia (100%)
